# An Updated Narrative Review on the Therapeutic Potential of Resveratrol in the Treatment of Cancer

**DOI:** 10.1002/hsr2.71995

**Published:** 2026-03-02

**Authors:** Mohammad Ashraful Islam, Al Amin, Md. Abdul Aziz, Md. Mahabubur Rahaman, Jakir Hossain, Jakir Ahmed Chowdhury, Mohammad Safiqul Islam

**Affiliations:** ^1^ Department of Pharmacy University of Asia Pacific Dhaka Bangladesh; ^2^ Department of Pharmaceutical Technology Faculty of Pharmacy, University of Dhaka Dhaka Bangladesh; ^3^ Department of Pharmacy, Faculty of Pharmacy University of Dhaka Dhaka Bangladesh; ^4^ Department of Pharmacy Noakhali Science and Technology University Noakhali Bangladesh; ^5^ Department of Computer Information Systems American International University Dhaka Bangladesh

**Keywords:** apoptosis, cancer, chemopreventive agent, prevention, resveratrol

## Abstract

**Background:**

Cancer is a significant global health challenge, affecting millions of people every year. Overall survival rates have improved over time, but some patients exhibit treatment resistance, prompting ongoing research to find suitable and effective sensitizers. Resveratrol, a natural polyphenolic compound, has demonstrated significant effects in inhibiting the growth and spread of cancer. However, the molecular mechanisms and applications of resveratrol remain unclear.

**Objective:**

In this updated narrative review, we aimed at discussing the role and related molecular mechanisms of resveratrol in various cancers and providing an overview of its therapeutic potential for the treatment and prevention of these cancers.

**Methodology:**

We conducted an extensive literature search across four major databases, including PubMed, Google Scholar, Web of Science, and ScienceDirect, to identify all relevant articles.

**Result:**

Resveratrol is a naturally occurring polyphenolic compound, specifically a stilbene, found in significant quantities in grapes, berries, peanuts, and various other plants. Resveratrol significantly contributes to the reduction of various human cancers, including those of the breast, cervix, thyroid, prostate, brain, skin, colon, bone, and ovarian cancers. Resveratrol has been shown to influence cell cycle arrest, activate apoptosis, and suppress metastasis, all of which are achieved through various signaling pathways. Additionally, several in vivo studies have shown promising results suggesting it may reduce tumor development and extend lifespan in animal cancer models, among other benefits.

**Conclusion:**

This review indicates that resveratrol could serve as a prototype for developing more efficacious and less toxic therapeutic agents for the treatment of various cancers.

## 1 Introduction

Naturally occurring polyphenolic compound resveratrol (3,5,4′‐trihydroxystilbene) is a stilbene found in substantial quantities in various plant sources, including red wine, grapes, berries, and peanuts [1]. Red and white wines had resveratrol levels of 14 and 0.1 mg/L, respectively [2]. For grape juice and whole grapes, which range from 0.05 to 0.5 mg/L, and up to 3.54 mg/L according to the report. Following the initial reports of anticancer activity in 1997, this drug has gained significant popularity [3]. Since then, this molecule has attracted the attention of researchers due to its significant roles in controlling inflammation, tumorigenesis, and cardiovascular protection, among others [2]. Resveratrol also promotes SIRT1‐dependent deacetylation of p53, which enhances cell survival and decreases the Michaelis constant of sirtuin1, which is a NAD‐dependent deacetylase protein encoded by SIRT1 gene, for both the acetylated substrate and NAD(+). By promoting Sir2, it also mimics calorie restriction in yeast, enhancing DNA stability and prolonging longevity by 70% in yeast [4].

Part of the French Paradox is that grapes are effective medicinal agents against cardiovascular diseases. Similarly, researchers have found that high doses of resveratrol substantially prolong the lifespan of mammals [2]. Dietary polyphenols, such as resveratrol and curcumin (diferuloylmethane, the main ingredient in turmeric), have exhibited antioxidant and anti‐inflammatory properties that can modulate the activation of NF‐κB to mitigate the adverse effects of oxidative stress [5]. Furthermore, COX‐1 and COX‐2 enzymes are suppressed by resveratrol [6]. Despite advances in technology and pharmaceuticals over the past two decades, cancer remains a worldwide health burden [7]. In addition to other specialized treatments, anticancer medication, surgery and radiation are also used to treat cancer. According to the previous study, lifestyle factors such as alcohol intake, obesity, environmental pollution and food additives account for 90 − 95% of all cancers, and defective genes cause the remaining 5 − 10% of cancers [8, 9].

For years, people have used herbs as dietary supplements or complementary therapies, which impact the signaling of cells. For instance, as an alternative therapy, resveratrol is being used for the treatment of different kinds of cancer. This drug has several therapeutic and preventive benefits against various types of cancer, according to numerous studies. Additionally, it has been widely envisioned as having utility for anticancer therapy when paired with other chemotherapeutic agents [10]. To explain the effects of resveratrol on various cancer cells, Aluyen et al. [11] evaluated three ways, which are the effects of resveratrol against cell apoptosis, proliferation, and inflammation. Similarly, a relatively recent study also concentrated on several mechanisms via which resveratrol functions, including transduction of signals by the growth factor β, apoptosis and inflammation, and growth factors and receptor tyrosine kinase‐linked signaling pathways [12].

While the mechanisms of resveratrol against specific cancer types have been reviewed in earlier studies [13, 14, 15], this review provides an updated and broader explanation of the effects and mechanism of action of resveratrol on a wide range of cancer types. We also discussed the latest advancements in resveratrol‐based drug delivery methods.

## 2 Methods

To identify and extract information on the role of resveratrol in cancer, we conducted a comprehensive literature search across four major databases: PubMed, Google Scholar, Web of Science, and ScienceDirect. Our last search was conducted on August 31, 2025, to identify the updated literature. We focused specifically on studies that examined the role of resveratrol in cancer, including its molecular mechanisms, experimental approaches, and clinical findings.

Studies meeting the following criteria were eligible for inclusion in our study: (i) investigated the anticancer effects or mechanisms of resveratrol, (ii) reported findings from pre‐clinical and clinical studies, (iii) were published in peer‐reviewed journals, and (iv) were published in English. We also manually searched for additional studies in the reference sections of the included studies.

Studies were excluded following some exclusion criteria: (i) focused on diseases other than cancer, (ii) were case reports, letters to the editor, abstracts, or editorials/commentaries, or (iii) were not peer‐reviewed or pre‐prints.

## 3 Resveratrol

### 3.1 Sources of Resveratrol

Grapes, wine, grape juice, peanuts, cocoa, and berries of *Vaccinium* species, including blueberries, bilberries, and cranberries, contain resveratrol. The grape cultivar, its origin, and susceptibility to fungus all affect the amount of resveratrol present in grape skins [1, 14, 15, 16]. A significant factor influencing a wine's resveratrol level is the length of time it ferments in contact with grape skins. White and rosé wines are often lower in resveratrol than red wines because the grape skins are removed early in the winemaking process [17]. As a result, it is exceedingly challenging to produce precise estimations of the resveratrol concentration in the hundreds of wines from wineries worldwide due to variations between wine varieties, vintages, and locations. However, the amount of resveratrol in wine is often minimal, erratic, and unpredictable. Resveratrol is a small component of the entire family of polyphenols found in grapes and wine [18].

*Trans*‐resveratrol‐3‐O‐β‐glucoside (*trans‐picked*) is the main form of resveratrol found in grapes and grape juice, and large amounts of resveratrol aglycones are found in wines, which are thought to be the result of the fermentation of sugar breakdown [19]. Significant levels of cis‐resveratrol are also present in many wines; this compound can be generated during fermentation or liberated from vaneferin's (resveratrol polymers) [20, 21]. Although red wine contains a comparatively high proportion of resveratrol, it also contains other polyphenols in far larger amounts [22].

### 3.2 Structure of Resveratrol

**Figure 1:**
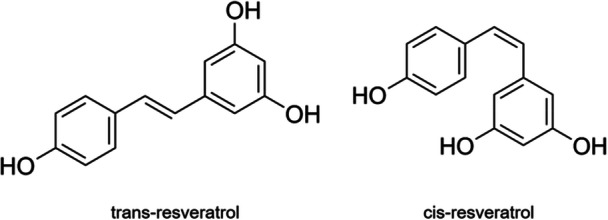
trans‐resveratrol and cis‐resveratrol chemical structure [19, 24].

The natural polyphenol resveratrol (3,5,4′‐Trihydroxystilbene) has a stilbene structure. Takaoka identified the chemical composition of this substance in 1940 after separating it from the roots of *Veratrum grandiflorum* [23]. It was also found in pharmaceutical products such as *manakka* or *darakchasava* [24]. The fundamental structure of the compound is comprised of a double styrene bond connecting two phenolic rings, resulting in the formation of 3,5,4′‐Trihydroxystilbene (molecular weight 228.25 g/mol). The isometric *cis*‐ and *trans*‐forms of resveratrol are due to the presence of this double bond in the structure (Figure 1).

## 4 Anticancer Effects of Resveratrol

Natural polyphenols are organic compounds that originate from plants and are identified by the presence of two or more phenol units in their structure [25]. Polyphenols have been the subject of numerous studies to investigate their potential health benefits, including protection against oxidative stress, diabetes, cardiovascular disease, neurodegenerative disorders, and aging [25, 26]. These polyphenols have a wide range of anticancer actions, including the induction of apoptosis, suppression of cell cycle events, and alteration of signaling pathways to eliminate cancer cells. They can modulate intracellular molecular levels to show antiangiogenic and anti‐metastatic effects [27, 28].

Resveratrol and grape extracts show that a significant number of prostate cancer cell lines experienced apoptosis when exposed to extracts [29]. Wine byproducts may aid in fighting against carcinogenesis, as evidenced by the pomace extract that remained following the production of wine significantly reduced the matrix metalloproteinases‐2 and −9 activities and showed a potent antiproliferative effect on human colon adenocarcinoma cells [30]. Additionally, grape juice phenolics dramatically reduced the rat model's ability to generate carcinogen‐induced DNA adducts [31], and prevented breast cancer cells from synthesizing DNA [32]. The anticancer properties of resveratrol are discussed in detail with their molecular mechanisms below (Table 1).

**Table 1:** Role of resveratrol in different cancers.

Cancer type	Potential targets	Mechanism of action
Glioblastoma	P38, ERK1/2 pathways (positive regulation); Autophagy‐related proteins (GFP‐LC3)	Resveratrol induces autophagy and cell death; effect via P38 and ERK1/2 signaling
Multiple myeloma	AMPK (↑ Thr172), mTOR (↓ Ser2448), 4EBP1 (↓Thr37/46), p70S6K (↓Thr389)	Resveratrol induces autophagy and apoptosis through AMPK activation and mTOR inhibition
Melanoma	PI3K/AKT/mTOR signaling; MAPK/ERK pathway	Resveratrol slows melanoma development via autophagy; reduces proliferation and increases differentiation by inhibiting PI3K/AKT/mTOR and MAPK/ERK
Colorectal cancer	AKT pathway, STAT3, p53, Bax, Bcl2, Cyclin D1	Resveratrol blocks AKT signaling, reduces cell growth, induces apoptosis, halts cell cycle
Ovarian cancer	eEF1A2, Akt, Caspase‐3/7/9	Resveratrol inhibits eEF1A2 activation, triggers apoptosis via caspase activation, blocks Akt signaling in PA‐1 cells
Breast cancer	HER2 (Δ16HER2), ERα, Proteasome, NF‐κB, mTORC1	Resveratrol acts as proteasome inhibitor, increases HER2, decreases ERα, affects NF‐κB and mTORC1 signaling, showing dual anti‐ and pro‐tumor effects
Pancreatic cancer	NF‐κB, ERK, STAT3 pathways	Resveratrol inhibits NF‐κB, ERK, and STAT3 activation, prevents tumor initiation and enhances chemotherapy sensitivity
Lung cancer	ROS‐mediated DNA damage, mitochondrial apoptotic pathway, STAT3, Akt, p53, Bax, Bcl‐2, Caspase‐3, MicroRNAs	Resveratrol induces apoptosis, autophagy, and senescence, regulates tumor macrophages, enhances chemotherapy response
Prostate cancer	Androgen receptor (AR), Steroid hormone response markers, PSA, AR‐negative pathways	Resveratrol inhibits AR expression, reduces tumor volume, induces apoptosis, decreases neovascularization and metastasis
Cervical cancer	Wnt (Wnt2, Wnt5a), Notch (Notch1, Notch2), STAT3, PIAS3	Resveratrol suppresses Wnt and Notch signaling, inhibits STAT3 phosphorylation, upregulates PIAS3, reducing cervical cancer spread
Chondrosarcoma	MMP2, MMP9, p38 MAPK, JNK, PI3K/MAPK pathway	Resveratrol inhibits MMP2/MMP9 expression via p38 and JNK, regulates PI3K/MAPK, reduces invasion and growth
Thyroid cancer	Ras/MAPK pathway, p53, SIRT1, PIP5Kγ	Resveratrol activates MAPK and p53, induces apoptosis in papillary and follicular thyroid cancer, improves iodide trapping and TSH secretion

### 4.1 Role of Resveratrol in Glioblastoma

Glioblastoma is the most prevalent and deadly primary brain tumor in adults [33]. The yearly incidence of this malignancy globally is around 7 cases per 100,000 people [34]. In the United States, more than 20,000 cases are identified annually. The mortality rate from gliomas is disproportionately high; more than 70% of individuals die within 2 years of diagnosis [35]. Approximately 65% of events of primary brain tumors are GBM, which is the most common and biologically aggressive kind, which is grade IV by the WHO classification system [36, 37]. A study assessed the autophagic level and cell proliferation of glioma cells after resveratrol treatment. In U373 glioma cells, resveratrol caused cell death and growth inhibition. Resveratrol treatment enhanced the number of GFP‐LC3‐labeled autophagosomes and the proportion of GFP‐labeled autophagosome‐containing glioma cells that maintained stable GFP‐LC3 expression. Moreover, the use of a P38 or ERK1/2 inhibitor pretreatment decreased the level of autophagy in glioma cells treated with resveratrol, indicating that the P38 and ERK1/2 pathways positively regulate resveratrol‐induced autophagy. The autophagy triggered by resveratrol did not involve the Akt/mTOR pathway. According to the findings, resveratrol induces autophagy in glioma cells, suggesting a potential anticancer effect [38].

### 4.2 Role of Resveratrol in Multiple Myeloma

**Figure 2:**
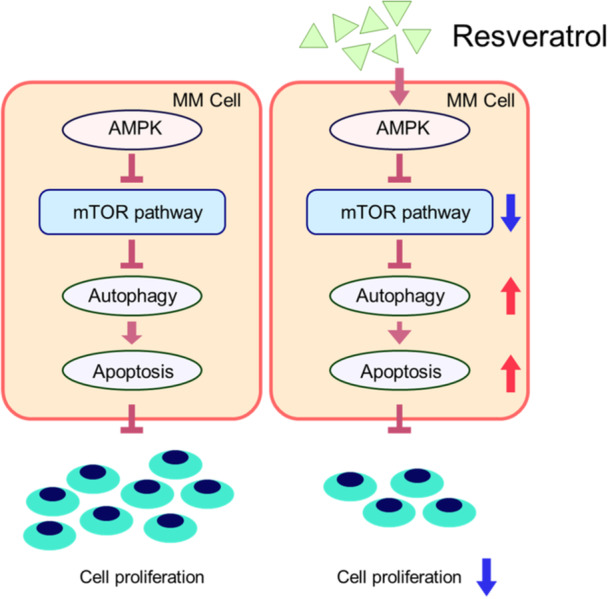
A functional model for resveratrol's activity on multiple myeloma cells. Resveratrol stimulates AMPK phosphorylation and inhibits mTOR phosphorylation, leading to autophagic apoptosis and a reduction in the development of multiple myeloma cells [40].

Myeloma, also known as multiple myeloma, is a malignancy of plasma cells. Resveratrol has shown anti‐multiple myeloma effects, which are thought to be mediated by inhibiting proliferation, inducing autophagy, and promoting cell death in multiple myeloma cells through the suppression of AMPK and mTOR signaling. The mTOR and AMPK pathways play a crucial role in regulating autophagy. Resveratrol decreases mTOR phosphorylation (Ser2448) and increases AMPK phosphorylation (Thr172) in certain solid tumors, resulting in anti‐cancer effects. Resveratrol increases the phosphorylation of AMPK (Thr172) and reduces the phosphorylation of mTOR (Ser2448) and its downstream components, 4EBP1 (Thr37/46) and p70S6K (Thr389), which shows reduced phosphorylation in multiple myeloma cells treated with resveratrol [39]. These findings indicate that the AMPK and mTOR pathways may mediate the effect of resveratrol on autophagy in multiple myeloma cells. Another study demonstrates that resveratrol induces both autophagy and apoptosis in these cells, with autophagy partially mediated by resveratrol‐induced apoptosis [40] (Figure 2).

### 4.3 Role of Resveratrol in Melanoma

Melanoma is a form of skin cancer that originates from melanocytes, the pigment‐producing cells [41]. It is sometimes referred to as malignant melanoma [42]. Most melanomas are typically found to occur in the skin; however, they can also happen in the mouth, intestines, or eyes (uveal melanoma) [43]. By inhibiting the PI3K/AKT/mTOR signaling pathway, resveratrol slows the development of melanoma in an autophagy‐dependent manner. Findings from past studies suggest that resveratrol may be able to prevent various cancers by regulating autophagy and inhibiting cellular growth [44]. A study states that by blocking the mitogen‐activated protein kinase kinase/ERK kinase pathway, resveratrol reduced proliferation, increased cellular differentiation, and improved melanin production in HT‐144 melanoma cells [45].

### 4.4 Role of Resveratrol in Colorectal Cancer

**Figure 3:**
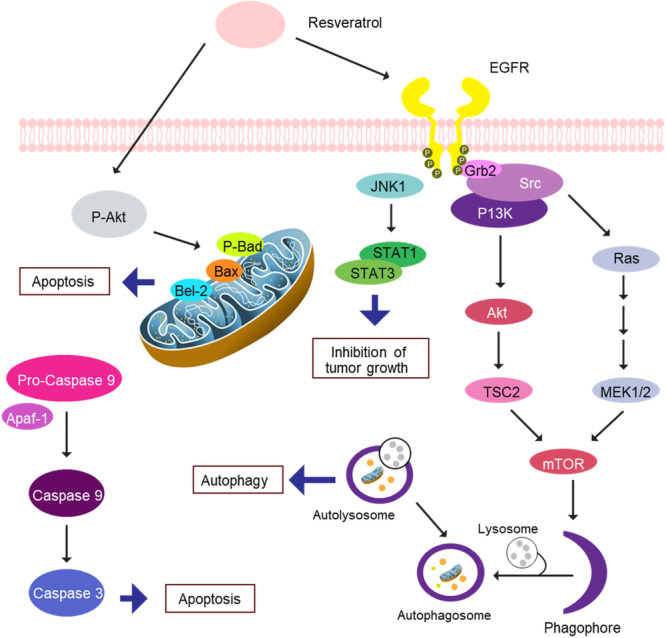
Targeting different signaling pathways using resveratrol in colorectal cancer [52]. (AKT, AKT serine/threonine kinase; Bcl2, BCL2 apoptosis regulator; STAT3, signal transducer and activator of transcription 3; p53, tumor protein p53; Bax, BCL2‐associated X; CDK, cyclin‐dependent kinase).

Colorectal cancer is the development of cancer from the parts of the large intestine, colon or rectum, often known as rectal, colon or intestinal cancer [46]. About 10% of all cancer cases worldwide are colon cancer, which is the third leading type of cancer [47]. There were 1.09 million new cases and 551,000 disease‐related deaths in 2018 [48]. Almost 65% of cases occur in developed countries, where it is more prevalent [49]. A variety of biological effects of resveratrol have been demonstrated, including anticancer activity, anti‐inflammatory effects, antifungal properties, antioxidant capabilities, and neuroprotective action [50]. It has been demonstrated that this drug inhibits the development of cancer cells through the AKT signaling pathway [51]. AKT inhibits p53 and Bax while upregulating Bcl2 and cyclin D1. It further promotes the activation of STAT3. As a result, AKT stimulates cell division, stops apoptosis, and advances the cell cycle. By blocking the activity of AKT and its downstream targets, resveratrol prevents cell growth, induces cell cycle termination, and promotes apoptosis (Figure 3) [53].

### 4.5 Role of Resveratrol in Ovarian Cancer

A cancer that develops in or on an ovary is known as ovarian cancer. It produces defective cells that may invade or spread to other bodily regions [54]. The liver, lungs, lymph nodes, and the lining of the abdomen are common places where cancer may progress [55]. Resveratrol's growth inhibitory effects on human PA‐1 cells for ovarian cancer: a possible molecular target to be considered is eEF1A2. Resveratrol pretreatment inhibits the growth of serum‐starved PA‐1 cells, which is triggered by insulin or serum. In addition, when insulin or serum is present, it induces apoptosis in PA‐1 cells by activating caspase‐9, −7, and −3. Resveratrol pretreatment inhibits the significant activation of eEF1A2 that was induced by stimulation of PA‐1 cells with insulin or serum. Additionally, in eEF1A2‐transfected NIH3T3 cells, resveratrol prevents the development of soft agar colonies induced by serum or insulin. According to an antibody array designed to measure protein kinase phosphorylation, insulin or serum administration can phosphorylate the Akt in PA‐1 cells. Resveratrol treatment slows down the expansion of the xenograft of a PA‐1 cell and the expression of eEF1A2 in athymic nude mice. This is accompanied by a decrease in bromodeoxyuridine positivity, a reduction in nuclear antigen expansion of proliferating cells, an increase in terminal deoxynucleotidyl transferase‐mediated dUTP nick end labeling and caspase‐3 staining, and a decrease in CD31 positivity. When combined, eEF1A2 may be regarded as a possible target for resveratrol's antiproliferative actions on PA‐1 ovarian cancer cells [56].

### 4.6 Role of Resveratrol in Breast Cancer

Breast cancer is the most common malignancy in women. Both the incidence and mortality rates are higher for this cancer. It is a multifactorial cancer with a variety of clinical and molecular characteristics [57, 58]. Resveratrol could potentially alter apoptosis via targeting multiple Notch pathway components such as Notch1, Jagged1 and Dll4 that are correlated to the progression of breast cancer [59]. Resveratrol treatment in Δ16HER2 mice not only predicts the onset of the tumor but also markedly increases tumor multiplicity, stimulating the development of more tumor masses per mouse. Furthermore, resveratrol therapy results in a marked upregulation of Δ16HER2 expression and a notable decrease in the level of ERα protein, both of which are consistent with its pro‐proliferative action. Resveratrol has been demonstrated to function as a strong proteasome inhibitor [60]. The data from both in vitro and in vivo experiments indicate that resveratrol can function as a proteasome inhibitor, as stated by Qureshi et al. [60] Nevertheless, resveratrol's capacity to inhibit the proteasome is a two‐edged tool. On the one hand, partial activity against inflammation by resveratrol has been demonstrated via blocking the activation of NF‐κB by the proteasome, which inhibits the activation of pro‐inflammatory cytokines and iNOS genes [61].

A simultaneous decrease in ERα is also associated with the rise in Δ16HER2 caused by resveratrol. As an ideal target for anti‐cancer therapy, ERα has a vital function in the initiation and progression of breast cancer [62]. However, the expression of ERα in cancers is dynamic and can change as the tumor progresses and after treatment [63]. One of the key mechanisms underlying the development of endocrine resistance is the loss of Erα [64, 65]. Many studies, both preclinical and clinical, indicate the presence of a significant interaction between HER2 and ERα, which often results in their inverse expression inside breast cancer. Remarkably, these bidirectional oscillations control the response to treatments that target HER2 and/or ER [66]. Consequently, it has been demonstrated that resistant breast cancer cells express HER2 more frequently than endocrine therapy‐sensitive cells do [67]. Resveratrol therapy mimics the development of endocrine resistance by inducing HER2 up‐regulation and ERα suppression. Again, resveratrol inhibits proteasome‐mediated ERα activation, hence counteracting its anti‐ERα effects [68]. ERα gene expression has been demonstrated to be repressed by several proteasome inhibitors [69].

**Figure 4:**
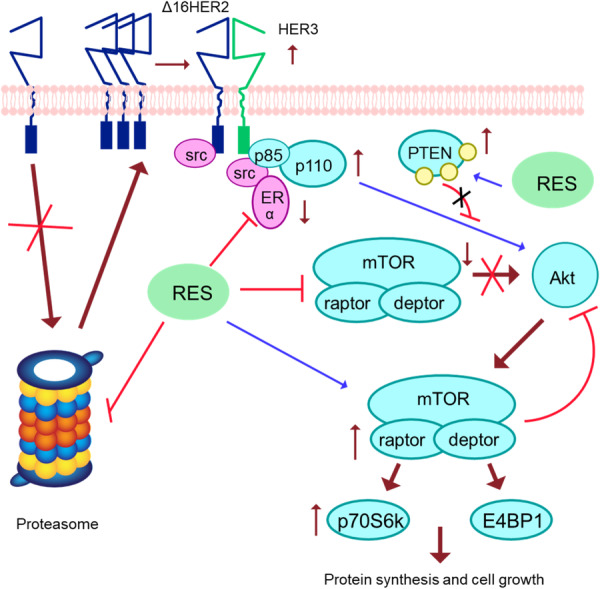
Resveratrol inhibits ERα and decreases the 20S proteasome's chymotrypsin‐like activity in HER2 + /ERα+ breast cancer [74].

Furthermore, it has been shown in a study that mTORC1 inhibition triggers autophagy and the Ubiquitin/Proteasome System [70]. Maintaining proteasome activity and mTOR signaling are inversely correlated. The activation of the mTORC1/p70S6K/p4EBP1 axis is the consequence of the resveratrol‐induced overexpression of Δ16HER2 [71]. Interestingly, prolonged life experiments have shown that rapamycin and resveratrol alone cannot extend the overall lifetime of mice as much as mTOR inhibition does, despite the purported anti‐aging effects of resveratrol [72]. This finding reinforces the link between resveratrol and the mTOR pathway even further and raises the possibility that aging‐related dysfunctions show preferential activation of the mTORC1 pathway [73]. The effects of resveratrol therapy are dependent on the inherent features of molecular cancer, functioning as a proteasome inhibitor and causing a multi‐target molecular rearrangement to promote tumor growth in the luminal B breast cancer subtype (Figure 4) [74].

### 4.7 Role of Resveratrol in Pancreatic Cancer

Pancreatic cancer starts when cells start to grow uncontrollably and accumulate into a tumor. These malignant cells are capable of spreading to other bodily regions [75]. The NF‐κB signaling system is regulated by resveratrol in various illnesses, including pancreatic cancer. NF‐κB activation occurs in precancerous lesions of PDAC, becoming highly noticeable after cerulenin activation, and resveratrol suppresses this phenomenon in vivo [76]. It is noteworthy that resveratrol treatment in vivo prevents pancreatic cancer from starting, possibly because resveratrol inhibits NF‐κB and the intended gene; activation of the NF‐κB signaling pathway in an in‐vitro study by exogenous addition of TNF‐α in an acinar 3D culture model accelerates the development of ductal‐like spheres (in vitro ADM/PanINs structures), a process that resveratrol stops [77]. Deleting IKB‐α, an NF‐κB inhibitor, reduced pancreatitis severity [78]. A study that found loss of IKKα, a promoter of NF‐κB, caused mice to develop spontaneous pancreatitis through the accumulation of p62 and endoplasmic reticulum stress [79].

**Figure 5:**
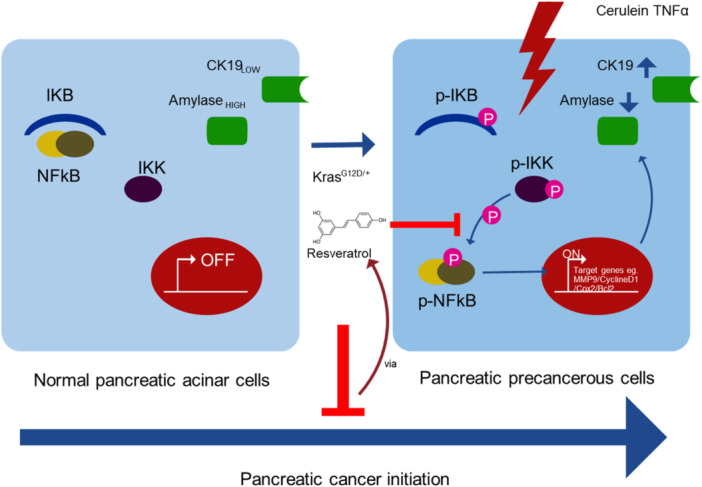
Resveratrol inhibits NF‐κB activation and delays the tumorigenesis of pancreatic cancer [82].

Furthermore, important molecular pathways in the onset and progression of pancreatic cancer include STAT3 and its activation, particularly in the treatment of patients who are resistant to chemotherapy [80]. It is interesting to note that ERK, STAT3, and NF‐κB have intricate and close relationships [81]. Moreover, research has shown that resveratrol suppresses the ERK and STAT3 signaling pathways to prevent both in vivo and in vitro malignant behavior of pancreatic cancer (Figure 5) [82].

### 4.8 Role of Resveratrol in Lung Cancer

Lung cancer, also known as lung carcinoma, is an uncontrolled cell growth in lung tissues that characterizes a malignant lung tumor. Roughly 98%–99% of all lung malignancies are carcinomas [83, 84]. Lung cancer claimed the lives of 1.8 million individuals worldwide in 2020, accounting for 2.2 million cases [85]. It is one of the primary factors contributing to death from cancer, and the most prevalent age of lung cancer diagnosis is 70 years old [86, 87]. Two kinds of lung cancer are known small‐cell lung cancer and non‐small‐cell lung cancer (NSCLC), which is one of the more prevalent types. The 5‐year survival rate for lung cancer is below 20%, despite recent advancements in treatment techniques such as intense radiation and/or chemotherapy [88]. By causing early senescence in NSCLC cells due to ROS‐mediated DNA damage, low‐dose resveratrol can prevent the development of lung cancer cells [89].

Trans‐resveratrol causes human lung adenocarcinoma epithelial cells to undergo apoptosis through a mitochondrial‐dependent mechanism [90]. Moreover, resveratrol protects lung cancer cells by regulating apoptosis and growth, binding the artificial or natural Egr‐1 promoter, and increasing GADD45α in A549 lung cancer cells [91]. By limiting the protumor activation of tumor‐associated macrophages in human lung cancer, resveratrol also successfully prevents lung cancer from progressing [92]. Additionally, resveratrol inhibits lymphangiogenesis and regulates M2 macrophage activation and differentiation in macrophages linked to a tumor, exhibiting anticancer and antimetastatic effects [93]. Furthermore, in lung cancer cells, resveratrol dose‐dependently reduces proliferation and triggers apoptosis via controlling p53, Bax, Bcl‐2, and cleaved caspase‐3 [94].

Resveratrol inhibits lung cancer both in vivo and in vitro in a dose‐dependent manner [95]. Within A549 cells, resveratrol uses STAT3 signaling to protect against cancer [96]. Additionally, by focusing on the Akt signaling pathway in NSCLC to limit tumor development, resveratrol inhibits glycolysis mediated by hexokinase II. Recent years have seen the development of several synthetic resveratrol analogs, such as 3,4,4′‐trihydroxy‐trans‐stilbene, which, in vitro, causes NSCLC cells to undergo autophagy and death [97]. Remarkably, miR‐200c increases the resveratrol susceptibility of H460 cells, probably through RECK expression [98]. The extended noncoding RNA AK001796 mediates resveratrol‐induced lung cancer cell growth suppression [99]. Furthermore, resveratrol also exhibits defensive properties against lung cancer by causing significant alterations in the A549 cell's microRNA expression profile [100].

Furthermore, resveratrol increases the effects of chemotherapy medications. For instance, in NSCLC cells resistant to gefitinib, resveratrol stimulates apoptosis, autophagy, and senescence, while working in concert with gefitinib to increase the intracellular concentration of the medication [101]. Both cisplatin and resveratrol together cause apoptosis in A549 cells by modifying autophagy [102]. Additionally, in A549 cells, resveratrol and arsenic trioxide act together to induce programmed cell death through ROS‐mediated endoplasmic reticulum stress and mitochondrial dysfunction [103]. Again, resveratrol was found to interact with other chemotherapeutic drugs, including erlotinib and etoposide in NSCLC [104].

### 4.9 Role of Resveratrol in Prostate Cancer

Prostate cancer is the second most frequent malignancy in males, although it often has a slow progression and increases with age [105]. Resveratrol significantly reduces prostate cancer in the transgenic adenocarcinoma mouse prostate (TRAMP) model [106]. By reducing the expression of the androgen receptor (AR) in the TRAMP model of prostate cancer, resveratrol prevents the growth of the disease. Resveratrol not only inhibits the expression of AR but also reduces the messenger RNA level of androgen‐responsive glandular kallikrein 11, an ortholog of the human prostate‐specific antigen [107]. In a xenograft model, resveratrol inhibited the expression of markers for steroid hormone response, which slowed the development of AR‐positive LNCaP tumors [108].

Oral resveratrol (30 mg/kg/day) administration after using AR‐negative PC‐3 human prostate cancer cell xenografts in the flank regions of mice decreased tumor volume, triggered apoptosis, and lessened tumor‐cell proliferation and neovascularization [109]. Resveratrol administered intraperitoneally (25 mg/kg/day) also reduced the tumor volume of PC‐3 cell xenografts in mice prostates [110]. Furthermore, resveratrol administered intraperitoneally (50 mg/kg/day) decreased tumor growth, progression, local invasion, and spontaneous metastasis in the orthotopic DU‐145 prostate cancer model [111].

### 4.10 Role of Resveratrol in Cervical Cancer

**Figure 6:**
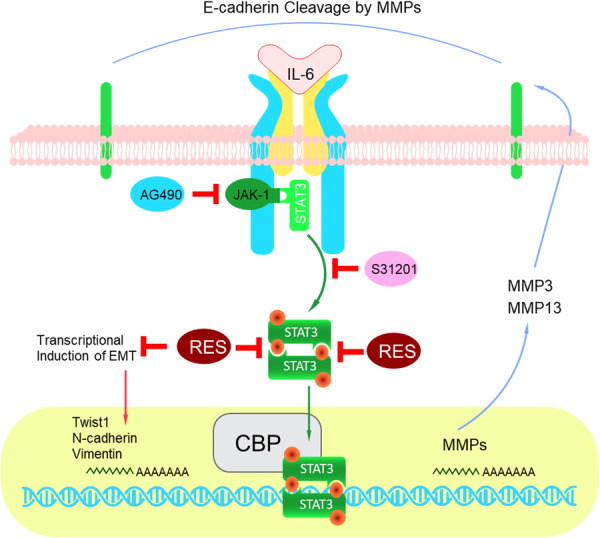
Resveratrol reduces the ability of cervical cancer cells to spread by deactivating the STAT3 pathway [118, 119].

In women, cervical cancer ranks as the fourth most common disease, accounting for 342,000 deaths in 2020 [85]. Resveratrol has been shown to have inhibitory effects on a variety of cancers, including squamous cell carcinomas and uterine cervical tumors' adenocarcinomas. [112, 113] Several signaling pathways linked to cancer are active in human cervical malignancies, and these pathways are used as therapeutic targets [114, 115]. Human cervical malignancies progress due to notch signaling in CD66+ cells [116]. Wnt inhibitory factor 1 is capable of blocking cervical cancer's development, invasion, and angiogenesis in vivo [117]. STAT3 signaling is constitutively active in cervical cancer and may contribute to the progression of cervical carcinogenesis caused by HPV16 [118]. Studies show that resveratrol's diverse biological actions are mirrored in its capacity to suppress the activation of signaling pathways linked to cancer [119]. A 100 μM of resveratrol effectively inhibited the development of the two cell lines and reduced the expression of Wnt2, Wnt5a, Notch1, and Notch2. While the transcriptional levels of STAT3 did not significantly differ between the HeLa and SiHa cells cultured normally and those treated with resveratrol, phosphorylation of STAT3 was clearly inhibited by resveratrol. This was accompanied by a distinct up‐regulation of PIAS3, suggesting that the main reasons for STAT3 inactivation can be these two occurrences (Figure 6) [119].

### 4.11 Role of Resveratrol in Chondrosarcoma

As a primary malignancy made up of cells originating from altered cartilage, chondrosarcoma is a kind of bone sarcoma [120]. Chondrosarcomas account for around 30% of all bone sarcomas. A chondrosarcoma can manifest at any age, unlike other primary bone sarcomas that often attack children and teenagers [121]. Resveratrol inhibits the development of chondrosarcoma cells mediated by MMP2 and MMP9 via the p38 kinase and JNK pathways [122]. It has also been observed that resveratrol inhibits p38 MAPK signaling. Resveratrol reduces the activation of MAPK in coronary artery smooth muscle [123, 124]. Also, by inducing apoptosis through affecting SIRT1 signaling, resveratrol shows antitumour activity [125].

### 4.12 Role of Resveratrol in Thyroid Cancer

Thyroid cancer is a kind of cancer in which cells grow improperly and may spread to other regions of the body. There may be edema or a lump in the neck as symptoms [126, 127]. Resveratrol has the potential to inhibit the growth of many cancers, including follicular and papillary thyroid cancer, by phosphorylating and activating the MAPK signal transduction pathway and increasing the amount of p53. It also affects thyroid activity by improving iodide trapping and favorably impacts metabolism by raising TSH secretion through the phosphatidylinositol‐4‐phosphate 5 kinase γ (PIP5Kγ) pathway and sirtuins activation [128].

In a study, resveratrol (1–10 µM) was applied to two cell lines of PTC (papillary thyroid carcinoma) and two cell lines of FTC (follicular thyroid carcinoma). The treatment resulted in nuclear translocation of MAPK, activation of the oncogene suppressor protein p53, serine phosphorylation of p53, and increased abundance of c‐fos, c‐jun, and p21 mRNAs. Additionally, resveratrol‐induced effects were blocked by inhibiting the MAPK pathway through H‐ras antisense transfection or PD 98059, a MAPK kinase inhibitor. Resveratrol‐induced p53‐specific inhibitor pifithrin‐alpha or transfection of p53 antisense oligonucleotides resulted in decreased levels of p53 and p21 expression in PTC and FTC cells [129].

## 5 Recent Advancements Using Resveratrol as an Anticancer Agent

Recent investigations have significantly advanced the development of nanotechnology‐driven delivery systems for resveratrol to address its inherent bioavailability challenges. Various nanodelivery mechanisms, such as liposomes, polymeric nanoparticles, lipid nanocarriers, micelles, and inorganic nanoparticles, have shown enhanced solubility, biocompatibility, and therapeutic effectiveness for resveratrol [130, 131]. These nanoformulations aim to tackle essential issues, including poor water solubility, rapid metabolic degradation, and light‐induced instability, which have limited resveratrol's clinical applicability [132]. Studies have indicated that nanoparticle‐encapsulated resveratrol exhibits improved anti‐cancer effects, including enhanced tumor targeting and reduced toxicity, with clinical trials reporting tumor regression alongside fewer adverse effects [133]. Innovative strategies under exploration include nanogels, hybrid nanoparticles, and synergistic treatments with immune checkpoint inhibitors [133].

A study by Soo et al. [134] described a dual nanoencapsulation approach that used liposomes containing both pristine‐ and cyclodextrin‐resveratrol complexes. This innovative method significantly enhanced cytotoxicity and achieved complete drug release in HT‐29 colon cancer cells compared to free resveratrol. In a subsequent study [135], researchers developed nanostructured lipid carriers (NLCs) that demonstrated a significant 15‐fold increase in the area under the curve and a five‐fold extension of the elimination half‐life, along with greater drug accumulation in the liver, lungs, and ovaries. Additionally, Perris et al. [136] conducted a thorough review of inorganic nanoparticles for resveratrol delivery, emphasizing their considerable potential against metastatic cancer. However, despite promising results from preclinical studies, no resveratrol‐based nanosystems have been approved for clinical use to date. This highlights the need to address regulatory frameworks, scalability challenges, and long‐term safety considerations of resveratrol [132, 133].

### 5.1 Limitations of Resveratrol as an Anticancer Agent

While resveratrol has been extensively studied for its anticancer properties, several limitations limit its clinical application. One primary reason is its low oral bioavailability, mainly because of its poor absorption in the digestive tract. In such cases, researchers have suggested approaches such as nanoformulations to improve their absorption and therapeutic efficacy [137, 138, 139]. Another limitation lies in the discrepancies between laboratory concentration and clinical concentration. Preclinical studies do cell‐based research on resveratrol at micromolar to millimolar levels, whereas in clinical settings (human), either from diet or supplements, concentrations rarely rise above nanomolar levels. This wide gap in achievable exposure makes it difficult to translate experimental findings into real‐world outcomes [138]. The effectiveness of resveratrol may also be influenced by interactions with natural hormones and other internal factors. For example, studies have shown that thyroid hormones can interfere with resveratrol's anticancer activity, which may partly explain the inconsistent results seen in clinical trials, where the expected outcomes are not always evident [138]. Again, differences in study design, sample, and outcome measures make it difficult to draw a consistent outcome of resveratrol's anticancer effect [139].

## 6 Conclusion and Future Direction

This review suggests that resveratrol may act as a possible anticancer agent through multiple biological mechanisms rather than relying on a single pathway. It has demonstrated its potential to modulate diverse oncological processes, including inducing apoptosis and autophagy, inhibiting cell growth, and decreasing the spread of cancer across a variety of cancer types, including glioblastoma, multiple myeloma, melanoma, colorectal, ovarian, breast, pancreatic, lung, prostate, cervical, chondrosarcoma, and thyroid cancers. These effects appear to result from the defined and coordinated modulation of several key signaling pathways, including PI3K/AKT/mTOR, ERK/MAPK, NF‐κB, STAT3, and SIRT1‐p53.

Future research studies should focus on high‐risk conditions where signaling mechanisms are significantly dysregulated, such as NF‐κB/STAT3‐dependent pancreatic cancer, EGFR‐TKI‐resistant NSCLC, and androgen receptor‐based prostate cancer. Inclusion of pharmacodynamic markers by targeting these pathways is also necessary to confirm resveratrol's efficacy. In lung cancer, for example, resveratrol combined with DNA‐damaging agents or EGFR inhibitors might be crucial, and outcomes could be assessed using markers such as p‐STAT3 and γH2AX. There is also evidence that resveratrol can influence the tumor microenvironment, suggesting its promising application alongside immunotherapies and necessitating assessment utilizing immune cell profiling. Moreover, varying doses of resveratrol may exert distinct effects, such as apoptosis and autophagy, underlining the importance of identifying effective doses. Clinical trials should concentrate on using biomarkers and include studies to confirm target engagement. Resveratrol could also serve as a starting point for developing more effective derivatives with greater therapeutic potential against cancer than resveratrol alone.

## Author Contributions

**Mohammad Ashraful Islam:** conceptualization (lead), writing – original draft (lead), writing – review and editing (equal). **Al Amin and Md. Mahbubur Rahaman:** visualization (equal), methodology (equal), writing – review and editing (supporting). **Md. Abdul Aziz and Jakir Hossain:** conceptualization (lead), methodology (lead), writing – review and editing (equal). **Jakir Ahmed Chowdhury and Mohammad Safiqul Islam:** conceptualization (lead), project administration (equal), writing – original draft (supporting), writing – review and editing (equal).

## Conflicts of Interest

The corresponding author, Mohammad Safiqul Islam, is a member of the editorial board, and he was not involved in any stage of decision‐making for this manuscript. The other authors declare no conflicts of interest.

## Transparency Statement

The lead author Mohammad Safiqul Islam affirms that this manuscript is an honest, accurate, and transparent account of the study being reported; that no important aspects of the study have been omitted; and that any discrepancies from the study as planned (and, if relevant, registered) have been explained.

## Data Availability

The data that support the findings of this study are available from the corresponding author upon reasonable request.
